# Pilot study on an innovative biosensor with a range of medical and surgical applications

**DOI:** 10.1186/s13104-018-3163-6

**Published:** 2018-01-29

**Authors:** P. Sains, K. S. Chana, V. Sridhar, M. S. Sajid

**Affiliations:** 1 Osney Thermo-fluids Laboratory, Department of Engineering Science, University of Oxford, Oxford, OX1 3PJ UK; 2 Department of General, Endoscopic and Laparoscopic Colorectal Surgery, Brighton & Sussex University Hospital NHS Trust, Eastern Rd, Brighton, East Sussex BN2 5BE UK

**Keywords:** Biosensor, Thermal product, Tissue density, Tissue conductivity

## Abstract

**Objectives:**

The objective of this article is to briefly outline the utilization of biosensors in medicine and surgery and present diagnostic efficacy of thermal product (TP) based biosensor.

**Results:**

The working principle of biosensor is based on measuring TP of a material in contact with the sensor. When an electrical square wave pulse of certain amplitude and duration is passed through TP based biosensor, the generated heat from its higher resistance will be dissipated and recorded by the sensor. As the surrounding material composition changes, the dissipated heat split between the sensor substrate and surrounding material changes which can be correlated to the change in TP of the material. For biological tissues, it is known that the thermal properties of tissues are quite different for different layers in the body and hence the heat absorbed will be different. The experiments were conducted on biological and non-biological tissues. For data acquisition software LabView 2014 (64-bit) was used and software used for post-processing was MATLAB R2015a (64-bit). The resulting graphs of TP from various materials (oil, water, saline, acetone) and biological tissue (porcine belly, porcine thigh layers and porcine abdominal viscera) expressed prominent deflections indicating diagnostic efficacy of TP based biosensor.

**Electronic supplementary material:**

The online version of this article (10.1186/s13104-018-3163-6) contains supplementary material, which is available to authorized users.

## Introduction

The use of biosensors in medicine has been reported for decades and has made huge impact to improve healthcare due to their capability of multiplex analysis [1–3]. The World population is faced with communicable and non-communicable ailments like HIV/AIDS, tuberculosis cardiovascular diseases, cancers and diabetes [4]. Diagnostic technologies enabled by biosensors have promising role in current and future medical management solutions due to their accuracy and rapidity. Detecting cancer in its early stages is beneficial as early intervention and treatment improves outcomes. Cancer biomarkers are the most valuable tools for cancer detection, accurate pretreatment staging, determining the response of cancer to treatment, and monitoring disease progression. Biosensors can be designed to detect cancer biomarkers such as, Prostate-specific antigen (ionic liquid carbon nano tubes, PDA molecules), Cancer antigen 125, proteomics and to determine drug effectiveness at various target sites [5–11]. Similarly, as per cancer diagnosis, the rapid and accurate diagnosis of infection and sepsis is always a priority to halt morbidity or mortality. The traditionally used culture plate to identify an organism and sensitivity testing are slow which led to the development relatively rapid polymerase chain reaction [12] but the peptide based biosensors or cytokine based biosensors offer even faster and the real-time diagnosis of infectious agents [13–18]. The development of biosensors such as acoustic wave biosensors [19], optical detection of sepsis markers by liquid crystal based biosensor [20], and new RAPPID has expedited the sepsis diagnosis [21]. C-reactive protein, an established tool to quantify sepsis can also be measured by biosensor with higher sensitivity [22]. Nucleic acid based biosensors such as gene chips, have played an important role in the presence or absence of the KRAS genetic mutation, a monoclonal antibody-based therapy used for colorectal cancer.

## Main text

### Rationale for TP based biosensor

The objective of this article is to report the development of novel TP-based biosensor with easier operationalization technique. Platinum thin film gauges are used in measuring heat transfer and temperature in many flow situations. The gauges are known to be highly sensitive and have a fast response time. The TP is an important parameter in the investigation of heat transfer and governs the rate at which heat is transferred to a gas, liquid or solid and has been used in turbine heat transfer studies. Initially the method was used for detecting contamination in liquids such as water in oil and aviation fuel.

The sensor’s working principle is based on measuring the TP of a material in contact with the sensor. The sensor contains thin film platinum gauges painted on an insulating disc such as a vitreous or ceramic substrate and when an electrical square wave pulse of certain amplitude and duration is passed through the sensor, the sensor’s temperature increases due to its high resistance. The heat generated is dissipated in the sensor substrate bottom and some is dissipated in the material surrounding the sensor. This will dictate the temperature recorded by the sensor. As the surrounding material composition changes, the dissipated heat split between the sensor substrate and surrounding material changes which can be correlated to the change in TP of the material [23] in contact and near the sensor. In the case of detecting biological tissues, it is known that the thermal properties of tissues are quite different for different layers in the body and hence the heat absorbed will be different. For example, González et al. [24] has reported the thermal signatures of the melanoma and normal skin tissue to be significantly different. The relation between heat transfer and thermal product is derived as follows:

The theory can be derived from the 1-dimensional transient heat conduction equation with a step change in heat input.

$$ \frac{{\varvec{\partial }^{2} \varvec{T}(\varvec{x} ,\varvec{t})}}{{\varvec{\partial x}^{2} }} = \frac{{\mathbf{1}}}{{ \propto (\varvec{x})}} \frac{{\varvec{\partial T}(\varvec{x},\varvec{t})}}{{\varvec{\partial t}}} $$

where **T** is temperature, **x** is distance within the substrate, **t** is time,

$$ {\text{where}}\; \propto = \frac{\varvec{k}}{{\varvec{\rho c}}} $$

$\varvec{k}$ is conductivity, $\varvec{\rho}$ is density and $\varvec{c}$ is specific heat capacity.

The analytical solution for a step function in temperature of this equation is

$$ \varvec{\dot{q}_{wall}} =(\varvec{{T}_{wall}}{\varvec{(t)}-\varvec{T_{0}}}) {\frac{{\sqrt {\varvec{\pi}}}}{\mathbf{2}}} {\frac{{\sqrt {\varvec{\rho ck}}}}{\sqrt{\mathbf{t}}}} $$

where ${\dot{\text{q}}}_{wall}$ is heat transfer rate, T_*wall*_ is wall temperature, *T*_0_ initial conditions

$$ \dot{\varvec{q}}_{{\varvec{wall}}} \propto \sqrt {\varvec{\rho ck}} $$

The sensor has a wide range of applications and currently is being actively used in non-healthcare fields for detecting contamination in oil, fuel or any liquid.

### Results

#### Preliminary feasibility studies of TP based biosensor on non-biological samples

**Fig. 1:**
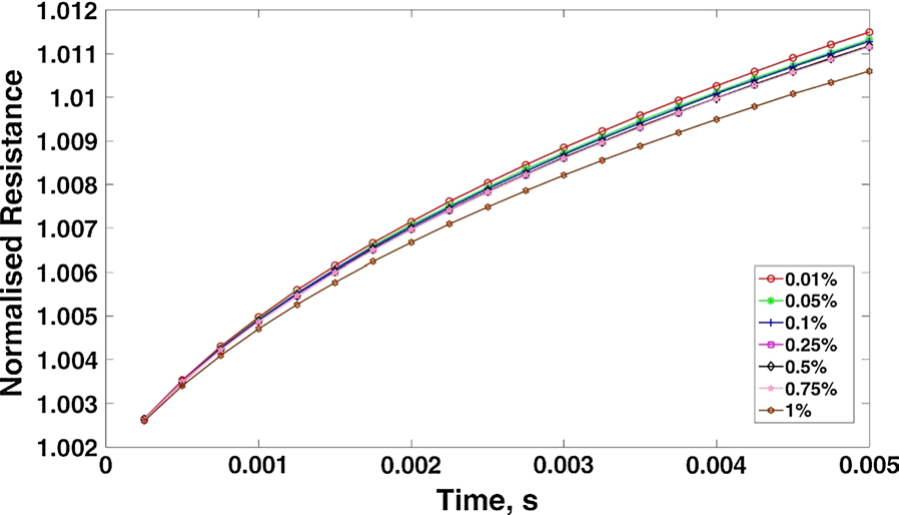
Thermal product curves for water in acetone

Initial tests were carried out with the TP sensor to look at contamination of water in acetone to judge the sensitivity of the biosensor. The experiments comprised of a pot with the sensor and an electronic device to send the electrical pulse and measure the response was made by Proxisense^®^ (Additional file 1: Figure S1a). The sensor had two thin film gauges made of platinum and these gauges were painted on a MACOR^®^ substrate (Additional file 1: Figure S1b). The fluids used in the experiments were acetone and water. A micro-litre syringe was used to supply the contamination at the required concentrations with the lowest concentration being 2 μL corresponding to 0.01% by volume for 20 mL of fluid. The device consists of a 24-bit analog to digital converter sampling at 4.8 kHz and can be configured to have varying pulse amplitude, width and frequency. In the tests carried out for contamination, the pulse amplitude was fixed at 5 V, the width was 5 ms and the frequency of pulsing was 2 s. The data from the device was acquired and then analysed using MATLAB^®^. Figure 1 shows the TP curves obtained from experiments with varying concentrations of water in acetone. One can note that the sensor is quite sensitive and is able to detect 0.01% of water in acetone (the measurements were repeated several times). The ascending voltage in the graph along y-axis represent varying TP of different layers. Since our preliminary studies showed that the sensor is quite sensitive and can detect small concentrations, a new sensor was built for use on animal tissues.

A biosensor using the TP technology has been developed using platinum thin film heat transfer gauges. The development of the sensor as a biosensor is a concept that adopted the above science which can distinguish between different forms of solid, liquid and gaseous matter. The authors’ hypothesis was that different types of biological tissue, fluids and gases would have their unique TP values, as per the non-biological tissues. Further hypotheses revealed that any deviation from the primary tissue, for example, would result in a different TP value. Therefore, diseased tissue may have a different TP than normal tissue due to differences in the metabolic rate and volume. The same would apply for liquids and gases where effectively, contamination has taken place by disease and its associated biomarkers. This is analogous to the use of the TP technology in the detection of oil contamination in engines.

The biosensor works on the principle of measuring the TP of the material, as the composition of the material in contact with the sensor changes the TP changes can be detected. The sensor can be used for real-time measurements due to its frequency response and has been demonstrated to be robust. Two sensor devices have been designed and constructed. One is for surface measurement (Additional file 2: Figure S2a). and the other, on a 7-gauge needle (Additional file 2: Figure S2b, S2c). Initial lab based tests were carried out to optimize the system for sensitivity and signal to noise ratio. The voltage was set to 5 V and the pulse width was 50 ms. The thermal product needle probe and the surface probe was traversed through biological tissue. As the layers were traversed the change in thermal product was recorded for each tissue layer.

#### Preliminary feasibility studies on biological tissue

**Fig. 2:**
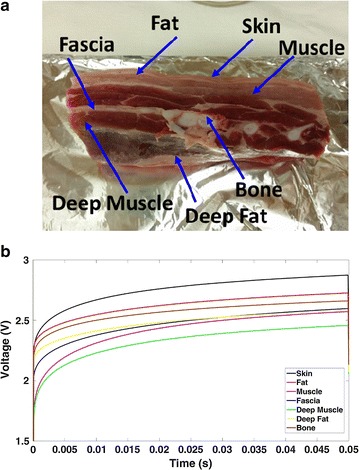
**a** Porcine sample pork belly. **b** Thermal product of different layers of porcine belly sample

Preliminary studies were carried on porcine samples. In these studies, the sensor was used to detect different tissues found in the porcine. In the first test, the sensor was used to classify different layers in pork belly such as skin, fat, muscle, fascia, deep muscle, deep fat and bone. Figure 2a shows a photograph of the pork belly sample used with different layers. The surface thermal product sensor was used to measure the TP of these different layers by stripping the base layer by layer. Figure 2b shows the thermal product curves clearly showing the different values of thermal product for each layer.

**Fig. 3:**
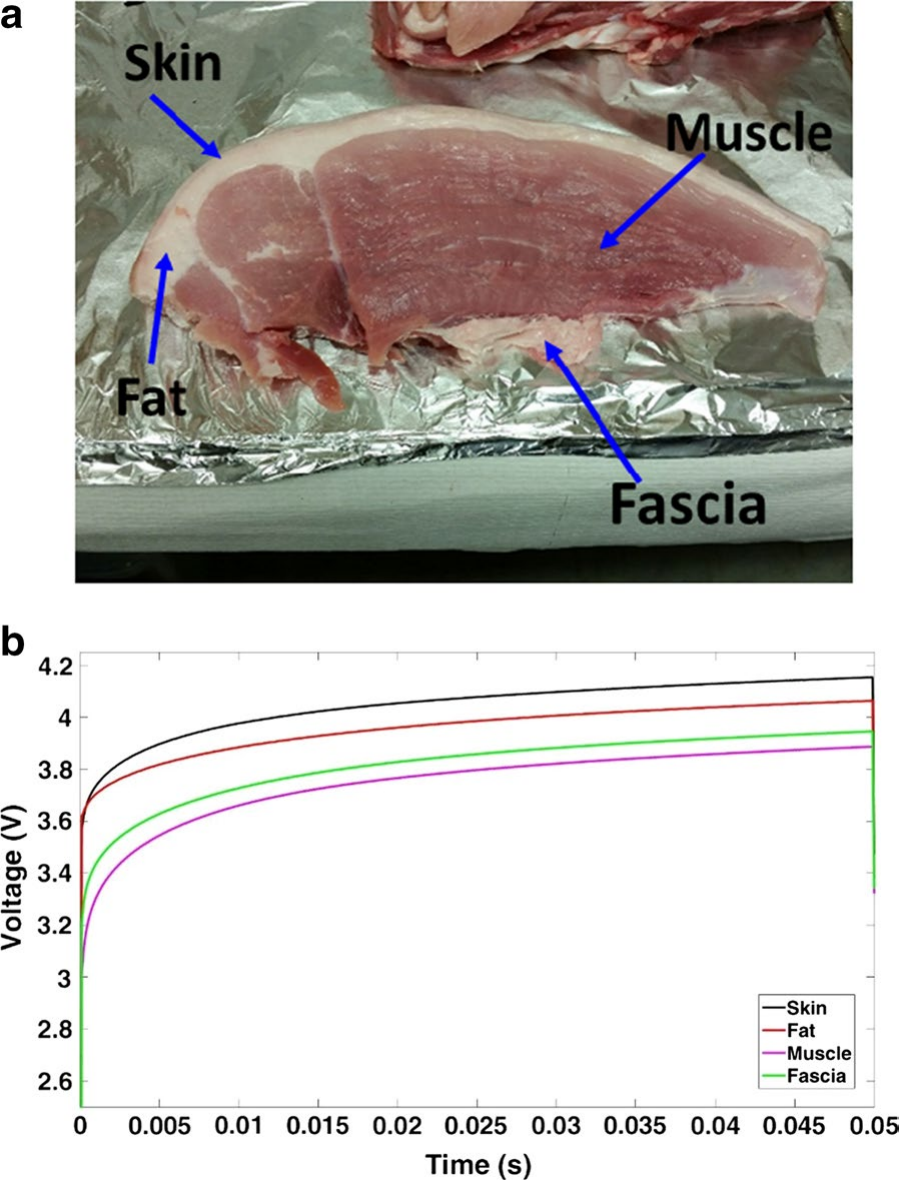
**a** Various layers of porcine thigh. **b** Thermal product curves of different layers in porcine thigh sample

In the second test, the sensor was used to detect similar tissues taken from a pork thigh. Figure 3a shows the thigh sample and again the surface sensor was used to measure the thermal product. Notably, the sensor has the ability to clearly distinguish the different layers using the measured thermal product (Fig. 3b).

In the final test, the sensor was used to detect different organ tissues such as liver, heart, lungs, aorta, oesophagus and thyroid from porcine samples (Additional file 3: Figure S3a, S3b) shows that the various organ samples are clearly distinguished through the thermal product curves.

## Implications

The TP based biosensor may have several advantages. This sensor device can be disposable and sterilizable. The sensors can be made of any size which opens up the opportunity of producing a hand-held device. The TP sensor device produces results in real-time excluding laboratory waiting time. This may possible aid the triage and appropriate referral to tertiary centers for specialist treatment. This may therefore, have an impact on clinical outcome through earlier triage and diagnosis as well as a positive effect on the healthcare cost. The TP sensor device, due to the advantage of its real-time analysis, may also be beneficial in surgical procedures. In the case of open oncological surgery, for example breast surgery, procedures are carried out to locally excise breast cancers and this device can be used at operating table to assure complete cancer excision. In the case of keyhole surgery the tactile differences between normal and diseased tissue are not conveyed through the instruments to the surgeon’s hands. The use of TP biosensors at the tip of the instruments may allow the differentiation between normal and diseased tissue to be made without the need for the haptic feedback. The concept can be further expanded by the use of the TP sensor needle to obtain biopsies, differentiate between tissues, differentiate between benign and malignant skin lesions, and contamination in body fluids. Further applications include the use of the thin film TP sensors on endoscopic instruments and surgical drains for the purpose of tissue recognition and the identification of early complications of surgery such as body cavity infected collections (abscesses). These are encouraging initial steps in the development of a biosensor technology that may has wider applications and noticeable impact on healthcare costs.

## Limitations

These initial experiments on TP based biosensor has shown its efficacy only on biological and non-biological tissue and not in real life clinical settings. Larger studies comparing its role with gold standard techniques such as histopathology or frozen section for resection margin clearance are mandatory. Furthermore, studies are required to quantify the effectiveness, efficacy in comparisons to other biosensors. The cost implication based studies are also required before recommending its wider medical and surgical applications.

## Additional files

**Additional file 1: Figure S1a.** The set up for intial test. **Figure S1b.** Platinum thin film gauges.

**Additional file 2: Figure S2a.** Surface probe to check TP on biological tissue. **Figure S2b.** Needle probe to check TP in deepr tissue. **Figure S2c.** Zoomed view of the tip of needle probe.

**Additional file 3: Figure S3a.** Porcine organ samples used for analysis. **Figure S3b.** Thermal product curves of different organs for porcine organ samples.

## Abbreviations

HIV: human immuno-deficiency virus
AIDS: acquired immunodeficiency syndrome
PDA: polydiacetylenes
K-RAS gene: Kirsten rat sarcoma gene
RAPPID: RAPPID is a fully-integrated, rapid multiplexed pathogen/biomarker immunodiagnostic device for rapid detection (< 15 min) of infectious diseases
