# Supplementary figures and images for: Pilot study on an innovative biosensor with a range of medical and surgical applications
Source: BMC Res Notes. 2018 Jan 29;11:81. doi: 10.1186/s13104-018-3163-6 (PMC5789537; doi:10.1186/s13104-018-3163-6)

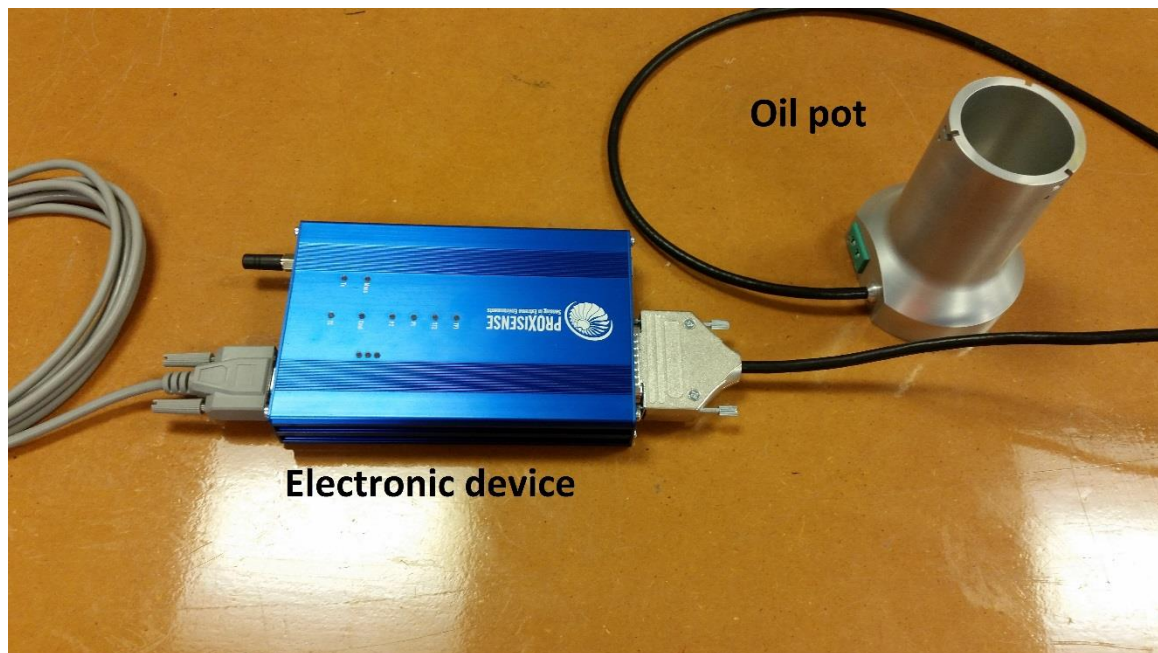

**Additional Figure 1a:**

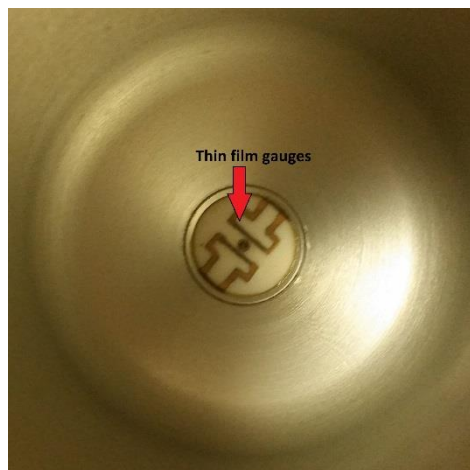

**Additional Figure 1b:**

Supplement: Supplementary file 1 — Additional file 1: Figure S1a. The set up for intial test. Figure S1b. Platinum thin film gauges. [file 13104_2018_3163_MOESM1_ESM.pdf]

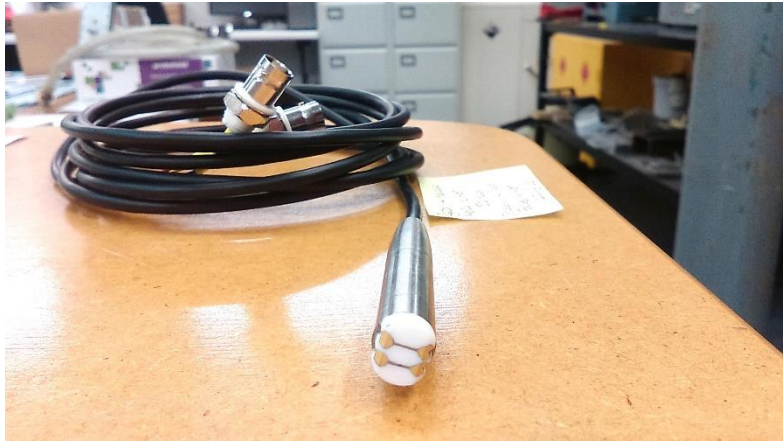

**Additional Figure 2a:**

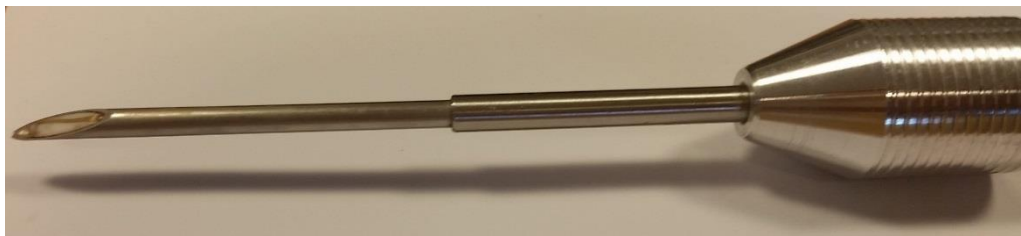

**Additional Figure 2b:**

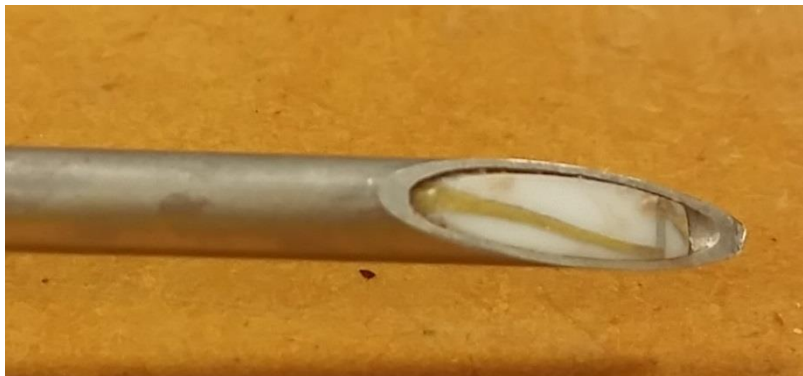

**Additional Figure 2c:**

Supplement: Supplementary file 2 — Additional file 2: Figure S2a. Surface probe to check TP on biological tissue. Figure S2b. Needle probe to check TP in deepr tissue. Figure S2c. Zoomed view of the tip of needle probe. [file 13104_2018_3163_MOESM2_ESM.pdf]

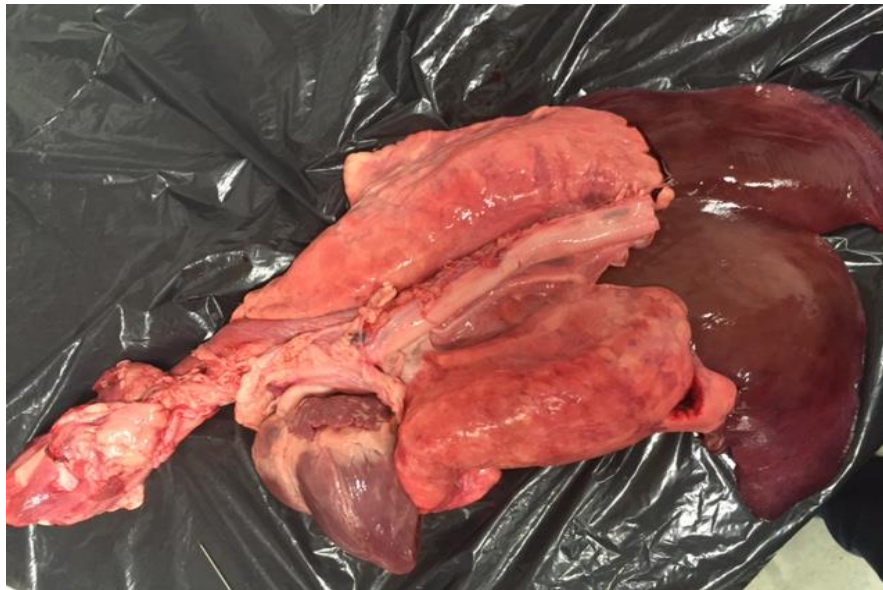

**Additional Figure 3a:**

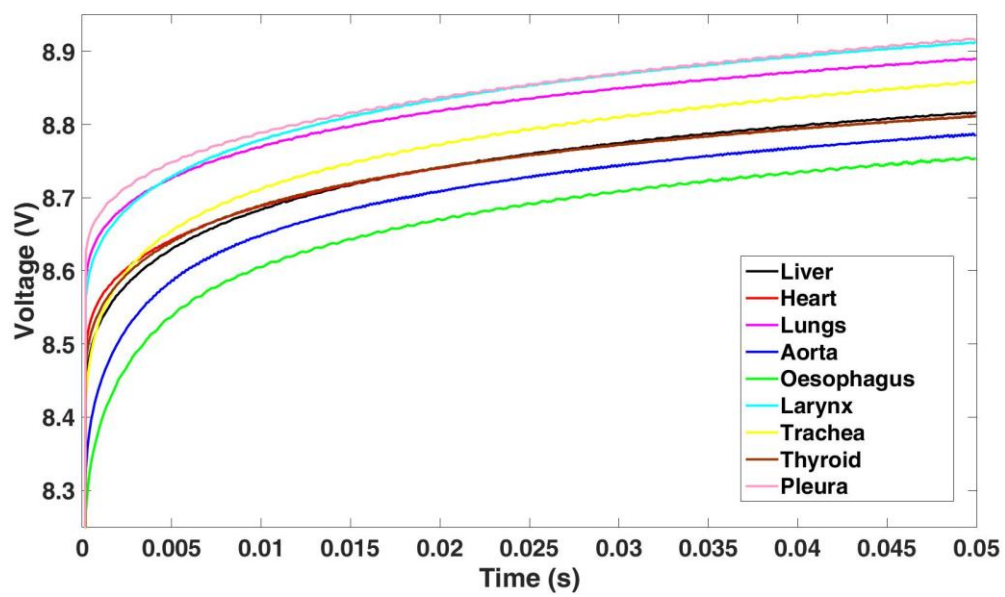

**Additional Figure 3b:**

Supplement: Supplementary file 3 — Additional file 3: Figure S3a. Porcine organ samples used for analysis. Figure S3b. Thermal product curves of different organs for porcine organ samples. [file 13104_2018_3163_MOESM3_ESM.pdf]
